# Supplementary figures and images for: Prognostic difference between surgery and external radiation in patients with stage I liver cancer based on competitive risk model and conditional survival rate
Source: PLoS One. 2024 Mar 28;19(3):e0298014. doi: 10.1371/journal.pone.0298014 (PMC10977706; doi:10.1371/journal.pone.0298014)

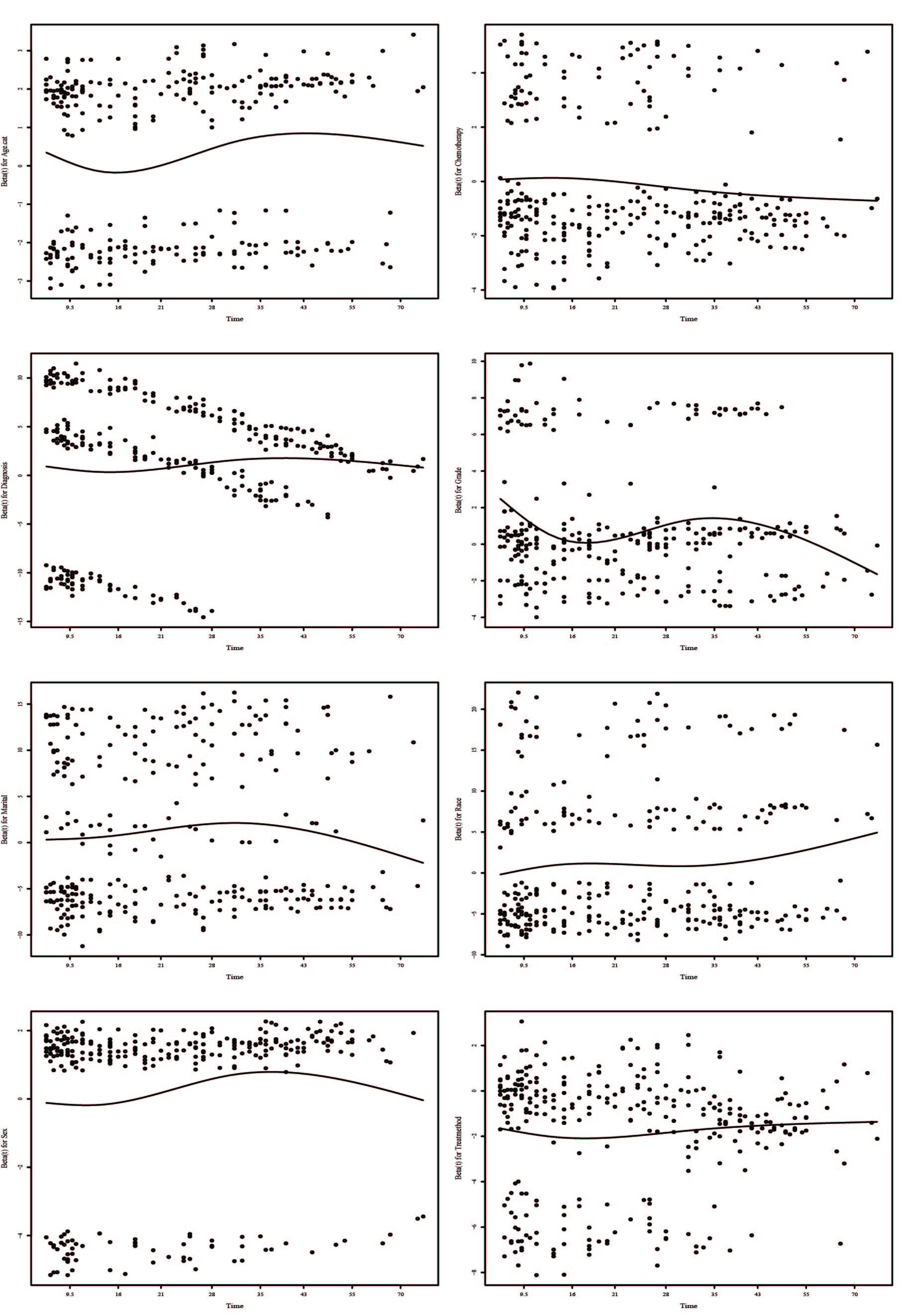

Supplement: S1 Fig — (JPG) [file pone.0298014.s002.jpg]

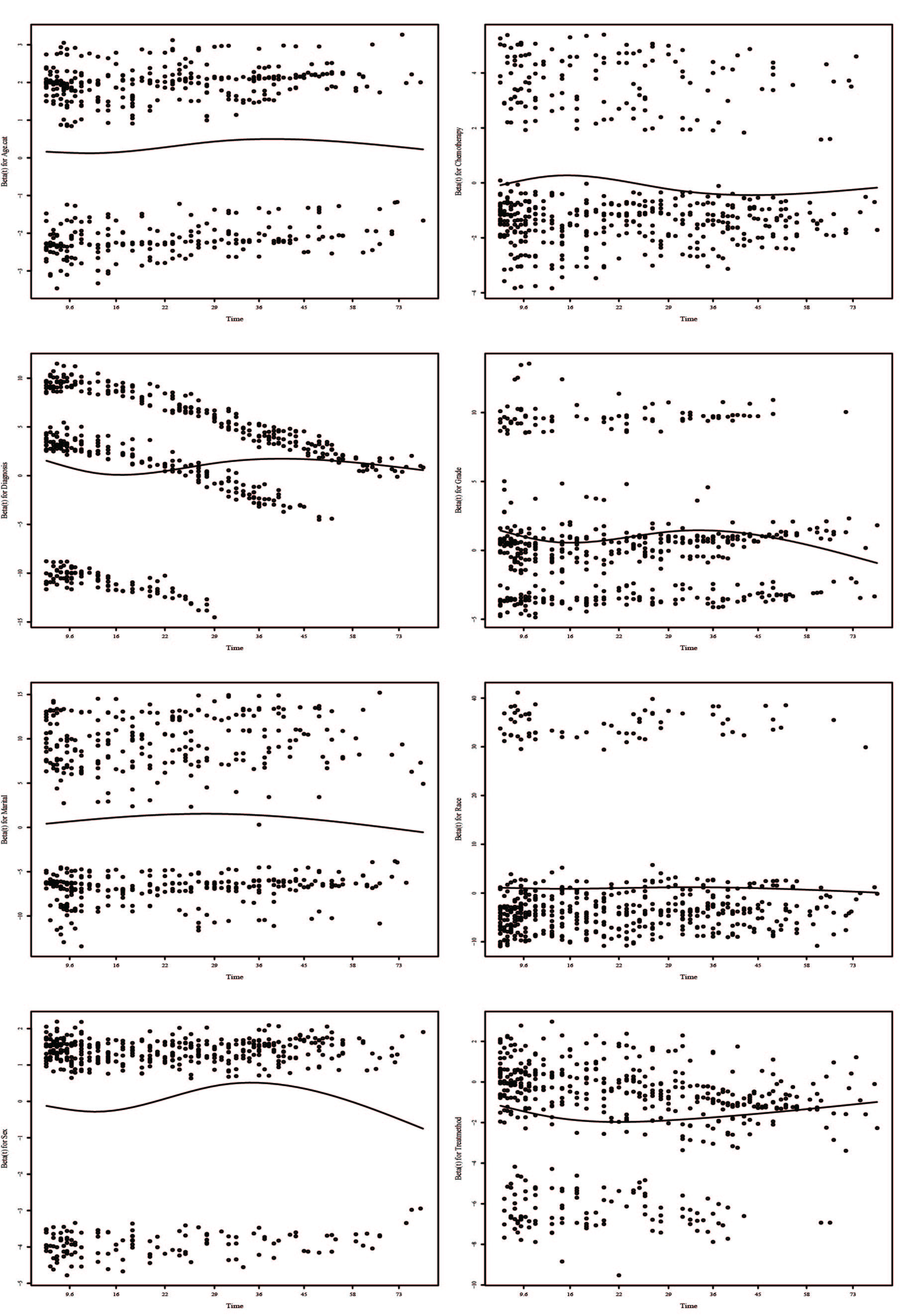

Supplement: S2 Fig — (JPG) [file pone.0298014.s003.jpg]

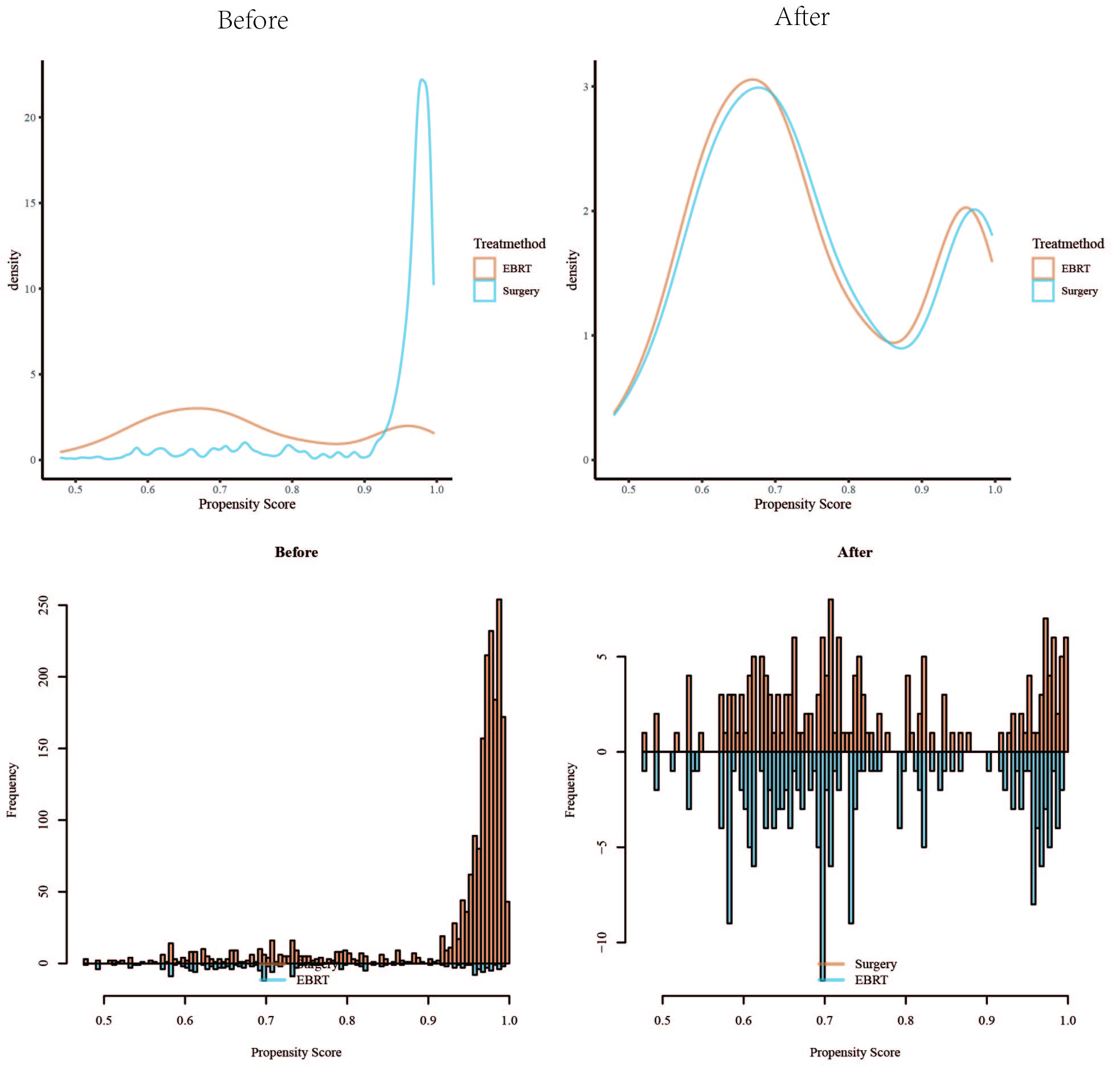

Supplement: S3 Fig — (JPG) [file pone.0298014.s004.jpg]
