# Supplementary material for: Prognostic difference between surgery and external radiation in patients with stage I liver cancer based on competitive risk model and conditional survival rate
Source: PLoS One. 2024 Mar 28;19(3):e0298014. doi: 10.1371/journal.pone.0298014 (PMC10977706; doi:10.1371/journal.pone.0298014)
Supplement: Attachment — Submitted filename: Response to Reviewers.doc [file pone.0298014.s005.doc]

Journal Requirements:

When submitting your revision, we need you to address these additional requirements.

1. Please ensure that your manuscript meets PLOS ONE's style requirements, including those for file naming. The PLOS ONE style templates can be found at

https://journals.plos.org/plosone/s/file?id=wjVg/PLOSOne_formatting_sample_main_body.pdf and

<https://journals.plos.org/plosone/s/file?id=ba62/PLOSOne_formatting_sample_title_authors_affiliations.pdf>

**Reply：**Thanks for the editor's suggestion, we have checked that our manuscript meet the requirements of PLOS ONE.

2. Thank you for stating the following financial disclosure:

“National Natural Science Foundation of China (81827805), National Key R&D Program of China (2018YFA0704100, 2018YFA0704104)”

Please state what role the funders took in the study.  If the funders had no role, please state: "The funders had no role in study design, data collection and analysis, decision to publish, or preparation of the manuscript."

If this statement is not correct you must amend it as needed.

Please include this amended Role of Funder statement in your cover letter; we will change the online submission form on your behalf.

**Reply：**Thanks for the editor's suggestion. We have added instructions to the Fund section as requested.

3. In your Data Availability statement, you have not specified where the minimal data set underlying the results described in your manuscript can be found. PLOS defines a study's minimal data set as the underlying data used to reach the conclusions drawn in the manuscript and any additional data required to replicate the reported study findings in their entirety. All PLOS journals require that the minimal data set be made fully available. For more information about our data policy, please see http://journals.plos.org/plosone/s/data-availability.

Upon re-submitting your revised manuscript, please upload your study’s minimal underlying data set as either Supporting Information files or to a stable, public repository and include the relevant URLs, DOIs, or accession numbers within your revised cover letter. For a list of acceptable repositories, please see http://journals.plos.org/plosone/s/data-availability#loc-recommended-repositories. Any potentially identifying patient information must be fully anonymized.

Important: If there are ethical or legal restrictions to sharing your data publicly, please explain these restrictions in detail. Please see our guidelines for more information on what we consider unacceptable restrictions to publicly sharing data: http://journals.plos.org/plosone/s/data-availability#loc-unacceptable-data-access-restrictions. Note that it is not acceptable for the authors to be the sole named individuals responsible for ensuring data access.

We will update your Data Availability statement to reflect the information you provide in your cover letter.

**Reply：**Thanks to the editor's suggestion, we have added a description of data acquisition in the corresponding section as“Raw data are made available on SEER(<https://seer.cancer.gov/>) database”.

4. We note that you have stated that you will provide repository information for your data at acceptance. Should your manuscript be accepted for publication, we will hold it until you provide the relevant accession numbers or DOIs necessary to access your data. If you wish to make changes to your Data Availability statement, please describe these changes in your cover letter and we will update your Data Availability statement to reflect the information you provide.

**Reply：**Thanks to the editor's suggestion, Raw data are made available on SEER (<https://seer.cancer.gov/>) database.

5. PLOS requires an ORCID iD for the corresponding author in Editorial Manager on papers submitted after December 6th, 2016. Please ensure that you have an ORCID iD and that it is validated in Editorial Manager. To do this, go to ‘Update my Information’ (in the upper left-hand corner of the main menu), and click on the Fetch/Validate link next to the ORCID field. This will take you to the ORCID site and allow you to create a new iD or authenticate a pre-existing iD in Editorial Manager. Please see the following video for instructions on linking an ORCID iD to your Editorial Manager account: <https://www.youtube.com/watch?v=_xcclfuvtxQ>

**Reply：**Thanks to the editor's suggestion, I have provided the ORCID iD.

6. Your ethics statement should only appear in the Methods section of your manuscript. If your ethics statement is written in any section besides the Methods, please delete it from any other section.

**Reply：**Thanks to the editor's suggestion, I have made corresponding modifications.

[Note: HTML markup is below. Please do not edit.]

Reviewers' comments:

Reviewer's Responses to Questions

**Comments to the Author**

1. Is the manuscript technically sound, and do the data support the conclusions?

The manuscript must describe a technically sound piece of scientific research with data that supports the conclusions. Experiments must have been conducted rigorously, with appropriate controls, replication, and sample sizes. The conclusions must be drawn appropriately based on the data presented.

Reviewer #1: Yes

Reviewer #2: Partly

2. Has the statistical analysis been performed appropriately and rigorously?

Reviewer #1: Yes

Reviewer #2: Yes

3. Have the authors made all data underlying the findings in their manuscript fully available?

The [PLOS Data policy](http://www.plosone.org/static/policies.action#sharing) requires authors to make all data underlying the findings described in their manuscript fully available without restriction, with rare exception (please refer to the Data Availability Statement in the manuscript PDF file). The data should be provided as part of the manuscript or its supporting information, or deposited to a public repository. For example, in addition to summary statistics, the data points behind means, medians and variance measures should be available. If there are restrictions on publicly sharing data—e.g. participant privacy or use of data from a third party—those must be specified.

Reviewer #1: Yes

Reviewer #2: No

4. Is the manuscript presented in an intelligible fashion and written in standard English?

PLOS ONE does not copyedit accepted manuscripts, so the language in submitted articles must be clear, correct, and unambiguous. Any typographical or grammatical errors should be corrected at revision, so please note any specific errors here.

Reviewer #1: Yes

Reviewer #2: Yes

5. Review Comments to the Author

Please use the space provided to explain your answers to the questions above. You may also include additional comments for the author, including concerns about dual publication, research ethics, or publication ethics. (Please upload your review as an attachment if it exceeds 20,000 characters)

Reviewer #1: it might be helpful for the clinician to specify which radiotherapy treatment schedules to propose for patients with early stage hepatocarcinoma. This is useful to guide the surgeon and radiation oncologist on which treatment schedules and techniques have achieved better results.

Reviewer #2: The authors performed an analysis of a large number of patients with liver cancer from SEER dataset assessing the role of surgery and radiotherapy in this subset.
The theme is of interest, but the analysis should consider some hints that could relate to actual treatment options.


Major revisions:
I suggest removing the marital status in the description of results.

**Reply：**Thanks for the reviewer's suggestion. Previous studies have found that marital status may have an impact on the incidence of liver cancer (PMID: 34774036, PMID: 33428602), so we included marital status in the analysis. At the same time, we also carried out a new analysis after removing the variable of marital status. In the multi-factor regression analysis, the main results and conclusions of this study were not affected without correcting marital status, that is Surgery was better than EBRT for patients with stage I liver cancer (as shown in the figure below). This not only proves the robustness of our conclusions, but also provides a basis for including marital status in our analysis.

The authors should add information about the surgical approach; regarding EBRT they should refer to the total dose and the delivery technique (3DCRT or VMAT or SBRT or Particle therapy). Regarding these variables, they should consider their impact and give some hints in the discussion section.

When considering the different time interval for surgery and the related different outcomes, the authors should provide considerations about it (different techniques?).

**Reply：**Thanks for the reviewer's suggestion. Based on the SEER database, this study found whether surgery and EBRT were disease-specific death factors in Patients with Stage I Liver Cancer through competitive risk model and correction of bias caused by competitive events, and found that the survival rate of surgery was higher than that of EBRT. Unfortunately, SEER did not record the specific information of surgery entry path, time and EBRT dose. Therefore, for patients who also received treatment, the impact of treatment interval and specific treatment methods on patient prognosis has not been studied. However, these variables are worth studying, so we have discussed them in the discussion section. Meanwhile, we are also conducting a prospective cohort study, which cannot be completed in a short time due to follow-up and other work in the cohort study. We are working hard to promote the above work and hope to carry out a series of research results in the future.

Also, some language tips:
- Line 69: replace “fatality” with “mortality”.
- Line 69: remove “management”.
- Line 72: replace “management” with “treatment”.
- Line 92: please provide definition of “DSS”.

**Reply：**Thanks for the reviewer's suggestion. I have made modifications according to the above requirements.

6. PLOS authors have the option to publish the peer review history of their article ([what does this mean?](https://journals.plos.org/plosone/s/editorial-and-peer-review-process#loc-peer-review-history)). If published, this will include your full peer review and any attached files.


If you choose “no”, your identity will remain anonymous but your review may still be made public.


**Do you want your identity to be public for this peer review?** For information about this choice, including consent withdrawal, please see our [Privacy Policy](https://www.plos.org/privacy-policy).

Reviewer #1: No

Reviewer #2: No
